# A call to combat the burden of Allergic Contact Dermatitis among children and adolescents with type 1 diabetes mellitus using medical adhesives: a cross-sectional observational study

**DOI:** 10.3389/fcdhc.2025.1665240

**Published:** 2025-10-22

**Authors:** Laila Alsuwaidi, Lana Kanj, Rasha Rowaiaee, Sara Kanj, Farah Otaki, Wafa Otypi, Mireille Bejjani

**Affiliations:** ^1^ College of Medicine, Mohammed Bin Rashid University of Medicine and Health Sciences (MBRU), Dubai Health, Dubai, United Arab Emirates; ^2^ Strategy and Institutional Excellence, Mohammed Bin Rashid University of Medicine and Health Sciences (MBRU), Dubai Health, Dubai, United Arab Emirates; ^3^ Department of Health Services Research, Care and Public Health Research Institute (CAPHRI), Faculty of Health, Medicine, and Life Sciences (FHML), Maastricht University, Maastricht, Netherlands; ^4^ Dubai Diabetes Center, Dubai Health, Dubai, United Arab Emirates

**Keywords:** type 1 diabetes mellitus, allergic contact dermatitis, medical adhesive, pediatric diabetes, adverse effects, United Arab Emirates, public health, sustainable development goals

## Abstract

**Background:**

The increasing prevalence of Type 1 Diabetes Mellitus (T1D) has led to the development of advanced technologies such as Continuous Glucose Monitors (CGMs) and insulin infusion pumps. These devices rely on adhesives to attached to the skin, which can trigger Allergic Contact Dermatitis (ACD) in some individuals. Despite their growing use, data on ACD prevalence among children/adolescents with T1D using adhesive-based medical devices in the United Arab Emirates (UAE) and the Gulf Cooperation Council (GCC) region remains limited. This study aimed to assess the prevalence of ACD in children/adolescents with T1D using CGMs in the UAE, and evaluate the association between device use and ACD. It also explored trends in immune-related comorbidities that could impact glycemic control.

**Methods:**

A cross-sectional observational study was conducted in collaboration with Dubai Diabetes Center (DDC). Medical records of 232 children/adolescents with T1D, receiving care at DDC between January 2020 and January 2023, were analyzed. Descriptive statistics were used to calculate proportions, and ACD prevalence was determined with a 95% Confidence Interval (CI) using Poisson distribution. Fisher’s exact test was applied to explore associations between categorical variables.

**Results:**

Among 232 study individuals, 87% (202 out of 232 individuals) used smart medical devices for glucose monitoring. Of these, 16 had a documented history of ACD, indicating a prevalence rate of 7.92% (95% CI: 4.6, 12.54). No statistically significant association was found between smart devices use and ACD development (p-value = 0.581). ACD prevalence was higher among females using adhesives (9.37%) compared to their male counterparts (6.6%).

**Conclusion:**

This study aligns with United Nations’ Sustainable Development Goals 3 and 4 by highlighting ACD prevalence among children/adolescents with T1D using CGMs in the UAE. It underscores the need for biomedical manufacturers to disclose adhesive chemical compositions to facilitate the development of safer alternatives. Additionally, healthcare professionals should be educated on dermatological risks associated with adhesive-based devices, enabling them to provide more comprehensive care and improve individual outcomes.

## Introduction

Type 1 Diabetes Mellitus (T1D) is a chronic autoimmune condition characterized by the immune-mediated destruction of insulin-producing pancreatic beta cells, resulting in insulin deficiency and persistent hyperglycemia (1). Effective management of T1D requires the daily administration of exogenous insulin and regular monitoring of blood glucose levels, tasks that can be particularly burdensome, especially for children/adolescents. This is of concern given that T1D most commonly presents in children between the ages of 4–7 and 10–14 years old (2). Despite advances in diabetes care, no cure currently exists, and the global incidence of T1D continues to rise. In 2021, approximately 1.5 million individuals under the age of 20 were diagnosed with T1D worldwide (3), including over 24,000 pediatric cases reported in the United Arab Emirates (UAE) alone during the same year (4).

The increasing prevalence of this metabolic disorder has driven the development of advanced medical technologies, including smart devices for Continuous Glucose Monitoring (CGM) and insulin infusion sets designated to deliver insulin in a controlled and sustained manner. These devices were introduced after extensive research in the early 2000’s (5). They significantly enhanced diabetes management as they support optimal glycemic control, improve individual adherence (particularly among pediatric populations), and reduce the risk of complications such as hypoglycemia and Diabetic Ketoacidosis (DKA). This is further supported by studies demonstrating a marked reduction in the incidence of DKA episodes after 6 to 12 months of consistent CGM use (4).

The use of adhesives is essential for securing glucose sensors and insulin infusion pumps to the skin. However, it is well established that these adhesives can lead to adverse skin reactions in individuals with T1D, most notably Allergic Contact Dermatitis (ACD) (6, 7). ACD is a delayed-type hypersensitivity reaction triggered by T-cell recognition of specific allergens, resulting in localized symptoms such as redness, swelling, and itching at the site of contact (8). Clinically, ACD can be differentiated by screening for specific features including cause, onset, location symptoms, and trigger, and/or through undergoing patch and/or histology testing. The gold standard method to diagnose ACD is patch testing (9, 10). The severity and frequency of the delayed-type hypersensitivity reactions can vary based on individual immune responses and levels of allergen exposure. Research has indicated that children/adolescents withT1D may be particularly vulnerable to ACD due to their age and relatively compromised immune system (8). Epidemiological data also reveal notable variability in ACD prevalence across regions. A 2022 study conducted in France reported that 33.8% of children/adolescents with T1D experienced ACD linked to medical adhesives (11), whereas a 2019 study from Italy found a significantly lower prevalence of 8.4% in a similar population (12).

Glucose sensors and insulin infusion pumps undergo clinical evaluation prior to regulatory approval and market release. However, due to the typically limited sample sizes in these trials particularly the underrepresentation of children/adolescents, ACD may be underestimated and not adequately recognized as a significant adverse effect (8). In the UAE, the prevalence of T1D among children and adolescents is of growing concern. According to the International Diabetes Federation (IDF), approximately 0.4 per 1,000 individuals aged 0–19 years are living with T1D in the UAE (13). Despite global awareness of ACD as a complication in children with T1D, region-specific data (particularly from the UAE and the broader Gulf Cooperation Council region (GCC) remain limited.

Measuring the prevalence of these often overlooked (at best, underestimated) undesired adverse effects, is crucial because they can result in reduced compliance and in turn poorer glycemic control. Accordingly, understanding the true burden of disease of adhesive-associated ACD, among children/adolescents with T1D in UAE, compounded with a holistic cost-to-benefit ratio analysis of using skin adhesives (for glucose sensors and/or insulin infusion pumps) needs to be attained, and will contribute to sustainable development in general, and in the attainment of select United Nations (UN)- Sustainable Development Goals (SDGs) more specifically. These adverse effects may constitute enough reason for advising individuals with T1D to revert to manual insulin injections and/or glucometers. At the very least, from a individual-centric perspective on care which is a priority in the health sector in Dubai, UAE in general and within Dubai Health more specifically, and in alignment with SDG 3: Good Health and Wellbeing (14, 15), fully understanding the actual risk of the respective adverse effects will empower individuals and their families to make informed decisions, maximizing effective shared decision-making between individuals and their physicians (16, 17). Generating this knowledge could also, in alignment with SDG 4: Quality Education, inform decisions related to health professions’ education to work towards better preparing relevant care providers for the prevention, diagnosis, and/or treatment of those adverse effects. Moreover, data on the association between current adhesive glycemic devices and ACD could prompt development of alternative adhesives with a lower risk of causing ACD.

The objective of the present study is to determine the prevalence of ACD among children/adolescents with T1D who use glucose sensors and/or insulin infusion sets for diabetes management in the UAE. Additionally, the study aims to evaluate the association between the use of these smart devices and the occurrence of ACD in this population. It also explores potential trends in immune-related conditions (such as allergies and comorbidities) that may influence glycemic control, comparing outcomes between them using smart devices and those managing their condition through traditional manual approaches.

## Methods

### Context of the study

UAE is a multi-cultural, multi-ethnic setting in GCC, rooted in Islamic values and Arabian traditions. UAE is a constitutional federation of seven emirates. Abu Dhabi city is the capital of the UAE federation, and Dubai is the largest and most populous of the seven emirates (16). UAE’s unique environmental and epidemiological landscape may contribute to the development of skin-related complications among individuals with chronic conditions. The high prevalence of atopic disorders in the region such as asthma, allergic rhinitis, and eczema could be indicative of the population’s heightened skin sensitivity and immune reactivity (18, 19). Additionally, UAE’s hot and humid climate can exacerbate skin barrier dysfunction, particularly in children with pre-existing atopic tendencies (20). In this context, the frequent and prolonged use of adhesive medical devices, such as continuous glucose monitors and insulin pumps, introduces a potential risk for skin sensitization and allergic reactions (21).

### Research design

This study relied on a cross-sectional observational design. The study was conducted according to the Strengthening the Reporting of Observational Studies in Epidemiology (STROBE) guidelines. UAE does not have a national registry that would have enabled the accurate assessment of T1D incidence and prevalence, which is also the case in several other countries. There exists, however, a health center that offers specialized care for individuals with T1D in Dubai, UAE, namely: Dubai Diabetes Center (DDC). This center is considered a center of excellence in enabling diabetes management by the International Diabetes Federation (21, 22), and constituted the source of data for the current study. In alignment with STROBE, consistency in data retrieval was assured through reliance on a preset data collection template. The ethical approval for this study was granted by the Mohammed Bin Rashid University institutional review board (MBRU-IRB-2023-35). All methods were performed following relevant ethical guidelines and regulations. Consent for publication was not applicable as the study involved retrieval of existing, anonymized, non-identifiable data.

### Study population

The study population included pediatric individuals [age ≤ 18 years, which aligns with global definitions of the pediatric population (23, 24)], with known T1D who received care at DDC between January 2020 and January 2023. The inclusion criteria specified that the pediatric individuals are UAE residents and have received care in DDC at least twice during the specified three-year timespan in order to detect any changes in their condition. Individuals with a single visit were excluded since having at least two data collection points is necessary to allow for measurement of changes in statuses, such as the development of ACD after exposure to external factors, including but not limited to adhesive devices. The diagnosis of T1D in DDC is made according to the American Diabetes Association (ADA) ‘Standards of Medical Care in Diabetes’ (5, 22, 25). Besides the exclusion of subjects who had a single visit to DDC during the 3 years, children/adolescents who had the Type 2 Diabetes Mellitus, and/or had missing data about T1D management method(s) were excluded from the study.

### Data collection

The medical records of all individuals in our study who received care at DDC are registered in Dubai Health’s Electronic Medical Records System, namely: ‘Salama’, including the physicians’ notes (through which ‘ the use of medical adhesive devices, as part of their diabetes management, can be traced). Data of all individuals enrolled in the study who matched the inclusion criteria was retrieved and recorded on a preset Microsoft Excel template. The data was anonymous, and no identifiers were recorded, all of which assured protection of individuals’ confidentiality.

The recorded data covered 10 variables. The demographics data included Medical Record Number (MRN), age, legal gender, and nationality. The data also included four dichotomous variables, inquiring whether, or not, the individual has ‘dermatitis’, allergies’, and/or ‘ACD’, and whether, or not, they use adhesives (as part of smart devices). The last two clinical variables included identifying individuals’ chronic conditions and also the result of glycosylated hemoglobin (HbA1c) test. Given that T1D is an autoimmune disease, it is commonly associated with other autoimmune diseases and conditions related to atopy (26, 27). Observing other chronic/recurrent immune-related conditions can be indicative of glycemic control. Therefore, enrolled individuals’ allergies and comorbidities were also recorded to keep track of any potential trend(s) in immune-related conditions. The latest HbA1c reading within the study timeframe was also collected to indicate the quality of glycemic control over the past two-three months. The latest International Society for Pediatric and Adolescent Diabetes (ISPAD) Clinical Practice Consensus Guidelines 2024 recommend an HbA1c target of ≤6.5% (48 mmol/mol) for youth using advanced diabetes technologies, assuming the populations have access to sufficiently skilled specialized healthcare professional service adept in diabetes education (28, 29). Relevantly, it is worth noting that the landscape of glycemic control of children and adolescents with T1D aged less than 18 years in the UAE is unknown, potentially limiting decisions around individual care, health system planning, and/or efforts around advocacy (28).

In relation to the diagnosis of adhesive-associated ACD, it was determined based on the presence of symptoms such as erythema, pruritus, rash, vesicles, blisters, and/or swelling at the adhesive site. These symptoms were noted and reported by the individuals to the diabetes specialist at DDC, who then confirmed the diagnosis of ACD. ACD can be confused with other types of dermatitis, such as Irritant Contact Dermatitis (ICD), atopic dermatitis, and/or seborrheic dermatitis.

**Figure 1:**
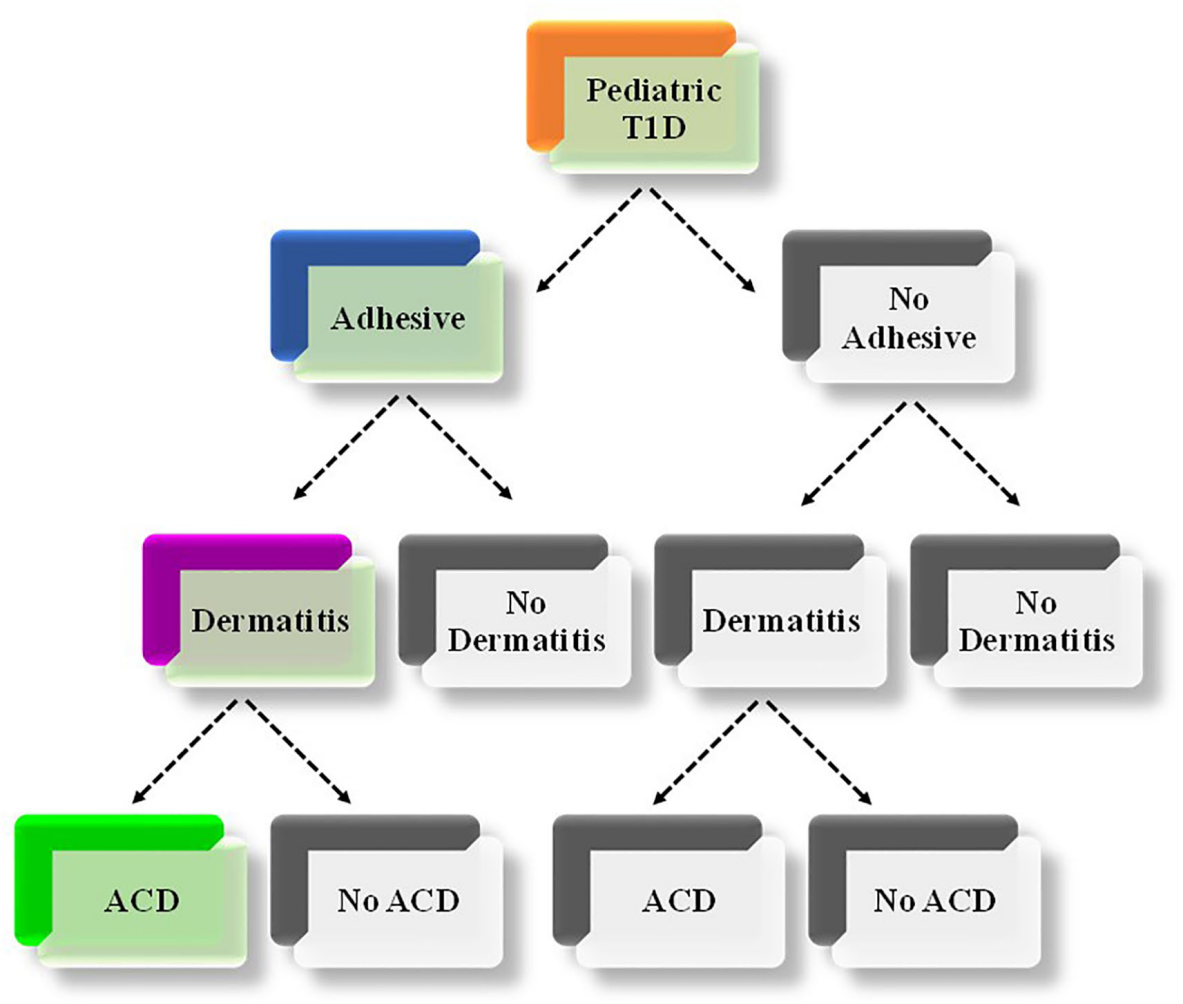
Illustration of the combination of relevant characteristics of the included study individuals. The thread depicted in colored boxes (Orange, blue and green) refer to the combination of characteristics that determine the focus of the current study (i.e., adhesive-associated ACD among children/adolescents with T1D). T1D, Type 1 Diabetes Mellitus; ACD, Allergic Contact Dermatitis.

As illustrated in **Figure 1**, individuals who were manually administering insulin injections and monitoring their glucose levels using blood glucose meters were classified as ‘no-adhesive use’. Those who used glucose sensors and/or insulin infusion pumps were classified as ‘adhesive use’. Individuals who had a dermatological condition diagnosis, including contact dermatitis, allergic contact dermatitis, dermatitis, allergic dermatitis, and/or dry skin dermatitis were labelled as ‘dermatitis’. The remaining individuals were labelled as ‘no dermatitis’. Among individuals with ‘dermatitis’, those who had ACD were grouped together, and the rest were classified as ‘not ACD’.

### Data analysis

Data was analyzed using Statistical Package for Social Sciences (SPSS), version 28.0.0.0. The descriptive analyses consisted of computing the proportions for all categorical variables, including ‘age’ (when categorized: 3-6, 7-10, 11-14, and 15–18 years), ‘legal gender’, and ‘nationality’; presence of ‘dermatitis’, ‘allergies’, and/or ‘ACD’; and use of adhesives, and of calculating the mean and standard deviation for ‘age’ as continuous variable. The prevalence of ACD who used adhesives among T1D individuals using adhesives was also calculated [95% Confidence Interval (CI) was calculated using Poisson distribution]. In terms of the inferential analysis, Fisher’s exact test was performed, a p-value ≤ 0.05 was designated to prove statistical significance.

## Results

### Characteristics of the study individuals

Data was extracted from medical records of 232 children/adolescents with T1D, out of which 196 (84.48%) were UAE nationals. The mean age of the study individuals was 12.72(± 3.87) years. Out of these individuals, 125 (53.88%) were males, and 202 (87%) used adhesive glucose sensors and/or adhesive insulin infusion pumps. Among the 202 study individuals who used adhesives, 96 were females and 106 were males. Approximately 15 (7.43%) of those who used adhesives were aged 3–6 years, 41 (20.30%) were aged 7–10 years, and 146 (72.27%) were aged 11–18 years (**Table 1**).

**Table 1:** Demographic characteristics of study individuals according to adhesive use.

Variable	Number of individuals	Adhesive	No adhesive
Nationality	232 (100)	202	30
UAE	196 (84.48)	175 (86.63)	21 (70.00)
Non- UAE	36 (15.52)	27 (13.37)	9 (30.00)
Sex	232 (100)	202	30
Male	125 (53.88)	106 (52.48)	19 (63.33)
Female	107 (46.12)	96 (47.52)	11 (36.67)
Age (Year)	232 (100)	202	30
3-6	18 (7.76)	15 (7.43)	3 (10.00)
7-10	45 (19.40)	41(20.30)	4 (13.33)
11-14	80 (34.48)	74 (36.63)	6 (20.00)
15-18	89 (38.36)	72(35.64)	17 (56.67)

A total of 232 individuals were included, with 202 in the adhesive group and 30 in the non-adhesive group. The majority of individuals enrolled in this study were of UAE nationality (84.48%), and this proportion was slightly higher in the adhesive group (86.63%) compared to the non-adhesive group (70.00%). Regarding sex distribution, males accounted for 53.88% of the total sample, with a greater proportion observed in the non-adhesive group (63.33%) than in the adhesive group (52.48%). Age distribution showed that the largest subgroup was 15–18 years (38.36%), followed by 11–14 years (34.48%), with smaller proportions in the 7–10 years (19.40%) and 3–6 years (7.76%) categories. Notably, more than half of the non-adhesive group (56.67%) were adolescents aged 15–18 years.

### Prevalence of dermatitis and Allergic Contact Dermatitis

Out of the 232 records studied, 102 (44%) study individuals were found to have a history of dermatitis, and among these, 18 (7.76%) had a diagnosis of ACD, with 16 (6.90%) of them using adhesive devices. The prevalence of ACD among females using adhesives was 9.37% (95% CI: 4.38, 17.05) and among males using adhesives was 6.6% (95% CI: 2.7, 13.13) (**Table 2**). Only two of the individuals diagnosed with ACD were not using adhesive glycemic devices. The overall prevalence of ACD among those using adhesives was 7.92% (95% CI: 4.6, 12.54), which was confirmed from the physicians’ notes to be an allergic reaction to the adhesives. None of the pediatric individuals in the youngest age group (3–6 years old) developed ACD. While the prevalence of ACD was 7.31% (95% CI: 1.53, 19.92) for individuals aged 7-10, and 10.81% (95% CI: 4.78, 20.19) for those aged 11–14 years. In those who were between 15 and 18 years old, the prevalence of ACD was 6.94% (95% CI: 2.29, 15.47). The mean age of individuals with ACD who used adhesives was 12.81 (± 3.124). Moreover,the prevalence of ACD among UAE nationals with T1D children/adolescents using adhesives was 8% (95% CI: 4.44, 13.06), while it was 7.41% (95% CI: 0.91, 24.29) for other nationalities. Out of the 18 subjects who developed ACD, 6 (33.33%) had pre-existing allergies to substances including kiwi, gluten, and Augmentin. Allergies were also reported by individuals without ACD. Out of the 214 individuals without ACD, 13 (6.07%) were allergic to eggs, Ibuprofen, penicillin, kiwi, and/or citrus.

**Table 2:** Distribution of dermatitis among study individuals according to adhesive use, sex, and age group.

Category	Dermatitis ACD	Dermatitis non-ACD	No Dermatitis	Total
Adhesive utilization				
Adhesive	16	75	111	202
No adhesive	2	9	19	30
**Total**	**18**	**84**	**130**	**232**
Sex				
Male	8	44	73	125
Female	10	40	57	107
**Total**	**18**	**84**	**130**	**232**
Age (Year)				
3-6	0	6	12	18
7-10	3	15	27	45
11-14	9	26	45	80
15-18	6	37	46	89
**Total**	**18**	**84**	**130**	**232**

The first section summarizes the occurrence of allergic contact dermatitis (ACD), non-ACD dermatitis, and no dermatitis in individuals exposed to adhesives (n = 202) compared to those not exposed (n = 30). A significantly higher proportion of ACD cases was observed in the adhesive group (n = 16) compared to the non-adhesive group (n = 2). The second section shows the distribution of dermatitis by sex, with relatively similar frequencies of ACD in males (n = 8) and females (n = 10). The third section presents age-related trends, where the frequency of ACD was highest in the 11–14 year (n = 9) and 15–18 year (n = 6) groups, while no cases were observed in the youngest group (3–6 years). In total, 232 individuals were included in the analysis, with 18 cases of ACD identified.

### Association between adhesive use and ACD development

**Figure 2:**
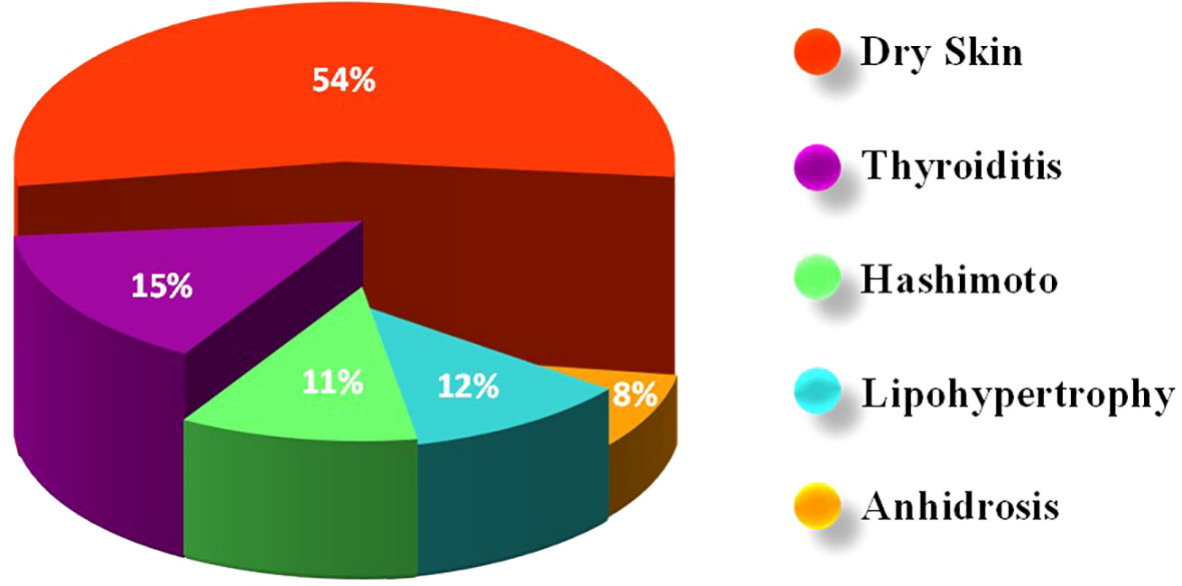
Distribution of comorbidities and complications among children/adolescents with Type 1 Diabetes Mellitus (T1D) with allergic contact dermatitis (ACD). The most frequently reported condition was dry skin (54%), followed by thyroiditis (15%), lipohypertrophy (12%), Hashimoto’s thyroiditis (11%), and anhidrosis (8%).

A two-sided asymptotic significance result was obtained from Fisher’s exact test that was used to analyze the significance between using adhesives and developing ACD. No statistically significant association was found between adhesives in glucose sensors and/or insulin infusion sets, and the development of ACD in the studied population (p=0.581). Most of the subjects, nine (56.25%) individuals who used adhesives and developed ACD reported dry skin in their problems’ list. Additionally, three individuals had Hashimoto’s disease, and three had thyroiditis. Anhidrosis had affected two individuals in the group. Other disorders such as celiac disease, thalassemia trait, vitamin B12 deficiency, hyperandrogenism, and obesity were also reported at very low percentages (**Figure 2**).

### Frequency distribution of HbA1c

**Figure 3:**
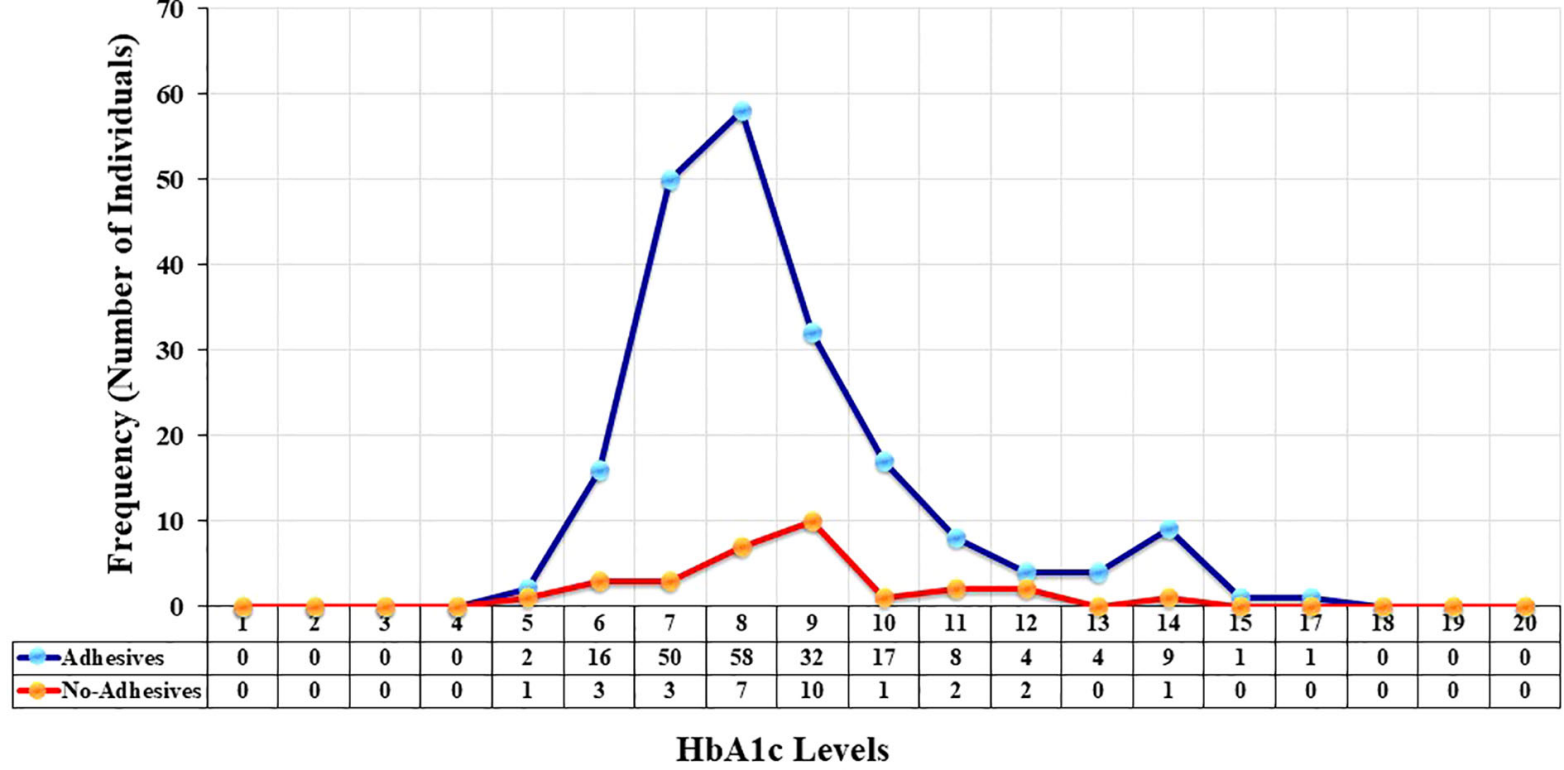
Frequency and distribution of glycated hemoglobin (HbA1c) levels among children/adolescents with Type 1 Diabetes Mellitus (T1D) using adhesives (blue line with blue markers) and individuals who do not use adhesives (red line with orange markers). The x-axis represents HbA1c levels, while the y-axis shows the total number of individuals.

The frequency distribution of individuals across different HBA1c levels with and without adhesives devices are shown in **Figure 3**. The x-axis represents the HbA1c level, while the y-axis indicates the frequency (number of individuals) within each category. In the adhesive group, HbA1c values showed a pronounced peak around 8% (64 mmol/mol), where the frequency reached 58 individuals. This suggests that the majority of the individuals in this group clustered around this HbA1c level. Beyond 8%, the frequency sharply declined, with progressively fewer individuals at higher HbA1c levels. In contrast, the non-adhesive group demonstrated a much lower overall frequency across all HbA1c levels. The highest concentration was observed around 9% (75 mmol/mol), though this peak only included about 10 individuals. Compared to the adhesive group, the distribution was flatter and less pronounced, indicating a broader spread of HbA1c levels but with fewer individuals at each point. Overall, among individuals who used adhesive devices, 46 (22.77%) had an HbA1c of 7.0% (53 mmol/mol) or less based on their most recent test. In contrast, out of those who did not use adhesive devices, only five (16.66%) had an HbA1c of 7.0% (53 mmol/mol) or less. This suggests differences in glycemic control profiles between the two groups.

## Discussion

To the best of the authors’ knowledge, this is the first study in the GCC to assess the prevalence of ACD among children/adolescents with T1D. In this study, 87% of individulas enrolled in this study were using glucose sensors and/or insulin infusion sets, showing that these smart devices are replacing conventional, manual insulin infusion sets, highlighting a clear shift from traditional insulin injections and glucose meters to advanced wearable technologies. The overall prevalence of ACD among the 202 participating pediatric T1D individuals using adhesives was found to be 7.92%.

### Comparison with international studies

Previous studies from outside the GCC have explored ACD prevalence in comparable populations (21, 30). A Belgian study reported a 3.8% prevalence among 1,036 users of Freestyle Libre glucose sensors, approximately half the rate observed in the current study. This lower prevalence might have been due to the limited scope of patch testing that the respective study relied on, which focused solely on isononyl acrylate, rather than screening for multiple potential allergens (31). Another five-year study (2015-2019), conducted in Denmark with 29 children/adolescents, found that 76.3% of them experienced skin reactions due to the adhesives (21). This high prevalence might have been affected by the research design of the respective study that relied on patch testing alone without screening of medical records. Similar to the findings of the current research work, a study conducted in Italy in 2019, including a total of 215 individuals with T1D using adhesives, reported a prevalence of 8.4% for ACD (12). Both the sample size and prevalence were comparable to those of the current study. The variability in reported ACD prevalence across studies may be attributed to differences in study populations, adhesive types, and ACD assessment methods (patch testing versus medical records). Nonetheless, it is established in the literature that ACD is a common and significant issue related to the use of glucose sensors and insulin infusion sets in children/adolescents with T1D (21). Also, allergic reactions and skin complications related to diabetes devices, such as ACD, tend to exert a substantial psychological burden on children and adolescents with T1D (32–35).

### Biological disease mechanisms

The current study population included children/adolescents with T1D aged 3 to 18 years. Notably, the majority of ACD cases (36.63%) occurred in the 11–14 year’s age group. While adhesive users were slightly more likely to be male (52.48% male versus 47.52% female), ACD was more prevalent among females (9 out of 16; 56.25%) relative to males (7 out of 16; 43.75%). This could be explained by the role of estrogen, which increases skin and mucosal permeability to allergens. All female individuals who developed ACD in this study were within the pubertal age range (11–18 years), further supporting the potential hormonal influence (10, 19, 22).

### Environmental disease mechanisms

There appeared to be no statistically significant association between adhesives in glucose sensors and/or insulin infusion sets, and the development of ACD. This finding may be attributed to the relatively small sample size, which was primarily due to the study’s emphasis on a particular subgroup of individuals in a single country in GCC. Although allergens unrelated to adhesives can also cause ACD (10, 36), it is noteworthy that the physician records consistently identified the onset of ACD at the site of adhesive application. This suggests that, contrary to the statistical, quantitative findings (which might have been skewed due to relatively small sample size), the adhesives themselves may indeed be the underlying cause of the observed ACD reactions.

Physicians’ notes frequently described the skin of ACD-affected individuals as ‘dry’, which may be reflective of the high prevalence of atopy (including eczema and asthma) in the UAE. This could, in part, be due to the high rate of consanguinity, particularly cousin marriages, within the population (18, 37, 38).The hot and humid climate may further exacerbate skin susceptibility to ACD development in response to adhesive devices. Two study individuals were found to have anhidrosis, highlighting a potential interaction between autoimmune responses and skin health. This aligns with existing evidence that T1D: an autoimmune disease, often coexists with other immune-medicated conditions, including dermatological and allergic diseases. The observed high prevalence of dermatitis and ACD in this cohort underscores the likely interplay between autoimmunity, atopy, and skin reactivity to external stimuli, such as medical adhesives. Consistent with this, comorbid autoimmune conditions such as Hashimoto’s thyroiditis were frequently reported among the individuals.

As previously noted, monitoring HbA1c level is a key indicator for effective diabetes management. According to ADA ‘Standards of Medical Care in Diabetes’, children/adolescents with T1D are advised to maintain HbA1c levels below 7% (53 mmol/mol) (39, 40). In this study, individuals using adhesive glycemic devices were more likely to have ‘achieved’ or ‘nearly achieved’ this target compared to non-users (41, 42). This supports the growing evidence that smart glucose-monitoring devices contribute to improved glycemic control by helping individuals maintain blood glucose levels within the optimal range. Consequently, the use of such devices is associated with better adherence, reduced glucose variability, and improved overall diabetes management compared to traditional methods. However, given the observed prevalence of ACD among children/adolescents with T1D in the UAE using adhesive-based devices, there is a clear need to address the adverse dermatological effects of these technologies.

### Public health implications

In alignment with the principles of sustainable development, particularly SDG 3: Good Health and Wellbeing- biomedical and pharmaceutical manufacturers are encouraged to prioritize innovation aimed at reducing the risk of ACD and to ensure that utilizing those devices results in more benefits than costs, equitably across the T1D population (irrespective of individuals allergy susceptibility). This may involve adopting hypoallergenic adhesives, redesigning device components to minimize skin contact, and/or incorporating more effective barrier sprays. Also, in alignment with SDG 4: Quality Education, it is essential to raise awareness among health professionals about the potential dermatological risks associated with these devices. Such knowledge, potential strategies for the treatment and prevention of such skin complications (43–46), should be embedded within formal education and professional development initiatives to ensure that clinicians are equipped to assess individuals holistically. This includes evaluating the risk–benefit balance of continuing with adhesive-based technologies versus recommending alternative methods, such as manual insulin injections or traditional glucometers, when necessary. Furthermore, in the context of shared decision-making (16, 17), healthcare providers should be well-informed and capable of educating individuals and their families about both the benefits and risks of smart devices. Empowering families through education will enable them to make informed choices not only about device usage but also about appropriate skincare practices to reduce the likelihood of ACD and ensure sustained diabetes control.

Implementing routine monitoring can further enhance treatment adherence and reduce the risk of skin irritation among pediatric individuals using adhesive-based devices. To better understand the prevalence of ACD and its association with medical adhesives in the context of T1D, larger-scale studies are required across the UAE. These studies should include a more diverse and representative sample of children/adolescents with T1D from multiple diabetes centers across the UAE. Expanding the research scope to include the broader GCC could provide deeper insights and strengthen the case for innovation in adhesive design. Such research could support the development of improved hypoallergenic adhesive technologies that minimize the risk of allergic reactions while maintaining device efficacy. In parallel, healthcare professionals play a vital role in advancing diabetes care by leading awareness campaigns and educational initiatives focused on the early identification and management of ACD related to medical adhesives.

A potential strategy to addressACD in children/adolescents with T1D is the use of patch test to identify specific allergens responsible for triggering symptoms. However, the feasibility of this approach is currently limited by a lack of transparency regarding the chemical composition of adhesive materials. This may uncover specificities of culprit substances that are often used in adhesives for diabetes medical devices, which include chemicals like acrylates [e.g., isobornyl acrylate (IBOA)] and cyanoacrylates. Other culprits include colophonium and its derivatives, as well as 2,2’-methylenebis (6-tert-butyl-4-methylphenol) monoacrylate. Repetitive exposure to those chemicals, compounded by the devices themselves, cause irritation and disruption of the skin barrier, contributing to skin lesions (30, 47–49). To overcome this hurdle, greater collaboration is needed between healthcare providers and biomedical and pharmaceutical companies to disclose and analyze the constituents and chemical combinations used in these products. Such collaboration would support the identification and development of safer, more tolerable alternatives for children with diabetes who are prone to allergic reactions. An additional concern commonly observed in individuals with T1D is dry skin, which is known to compromise the skin barrier and increase susceptibility to irritants, thereby increasing the risk of ACD (10). Healthcare providers should emphasize the importance of maintaining a consistent skincare routine, including the use of gentle, natural-sources moisturizers. Moreover, pediatric individuals may benefit from consultation with dermatologists to receive personalized, practical skincare regimens tailored to managing dry skin effectively and reducing the risk of ACD.

### Strengths of the current study

This study is the first to assess the prevalence of ACD among pediatric individualswith T1D and to examine its association with medical adhesives use, demographics, allergies, and comorbidities, both within the UAE specifically and the GCC more broadly. A key strength of the study lies in the use of data retrieved directly from Dubai Health’s consolidated Electronic Medical Records (EMR) system, which ensures comprehensive coverage across hospitals and continuity in tracking the study individuals journeys. These records, meticulously maintained by health and care professionals, offer reliable information on treatment modalities (e.g. use of adhesives versus manual injections) and provide detailed documentation of any diagnosed dermatitis, including ACD, from the initiation of each individuals’s medical file. This enhances diagnostic accuracy and reduces bias, enabling clear differentiation between ACD and other forms of dermatitis beyond the scope of this study. Another notable strength is the three-year study period, which contributes to the robustness of the findings by allowing for a more comprehensive view of individual experiences and outcomes over time. Moreover, the study was conducted at the DDC, a public healthcare facility that provides free care exclusively for UAE nationals and a preferred center for managing individuals with T1D among this population. Although the subjects studied belong to 15 different nationalities, the majority (84.48%) were UAE nationals. As such, the findings are highly generalizable to pediatric UAE nationals with T1D. However, it is important to note that UAE nationals comprise only 11.48% of the country’s total population as of 2023, with non-nationals representing the remaining 88.52% (24, 50).

### Limitations of the current study

A notable limitation of this study is its relatively small sample size, in general, and the number of ACD cases more specifically, which reduced the statistical power and may have limited the ability to detect significant associations. Additionally, data collected exclusively from children/adolescents with T1D at a single health center, introducing geographic constraints and limiting the generalizability of the findings to the broader UAE population. Despite these limitations, the study offers novel insights into a previously unexplored topic in both the UAE and the wider GCC. Although patch testing which is the gold standard method to diagnose ACD was not the means by which ACD was diagnosed in the current study, relying on the presence of symptoms as reported by the individuals to the specialist who in turn confirmed the diagnosis, might have reduced the possibility of false negatives associated with the limited scope/sensitivity of patch testing (51, 52). It is worth aiming for upcoming studies that involve diagnosing ACD to combine both mechanisms of screening in order to maximize sensitivity (minimizing false negatives).

### Future directions

The findings of the current study present valuable opportunities for future research. Investigating the demographic and clinical profiles of individuals more susceptible to ACD—such as age, sex, genetic predisposition, comorbidities, and existing allergic conditions—can help healthcare providers identify high-risk groups. Such information could support the development of tailored preventive strategies, personalized treatment plans, and targeted interventions to reduce the incidence and impact of ACD among vulnerable pediatric populations.

Future research studies must focus on developing non-allergic adhesives tailored for individuals prone to ACD, as well as implementing improved skin hydration practices and prescribing prophylactic moisturizers for individuals using adhesives. Expanding the study’s scope to encompass a more diverse sample, both geographically across the UAE and regionally within the GCC, would provide more comprehensive data on ACD prevalence and risk factors. Further exploring the relationship between atopy and the development of ACD in pediatric individuals using adhesives can deepen our understanding of the condition and inform targeted preventive measures. Given the shared environmental and lifestyle characteristics across GCC countries, regional studies can complement the current findings and contribute to the global literature on ACD management. Finally, future research should aim to define specific risk factors for ACD, evaluate the efficacy of preventive approaches, and assess the long-term impact of adhesive-related skin complications on glycemic control and overall quality of life. Addressing these knowledge gaps will be instrumental in improving treatment adherence, clinical outcomes, and individual well-being among children with T1D worldwide.

## Conclusion

ACD is an often-overlooked adhesives-associated dermatological complication in pediatric individuals with T1D who use smart devices, potentially hindering individual compliance and glycemic control. Despite the prevalence and burden of this array of adverse effects, there remains no clear, universal guidelines for preventing or at least managing them. The current study contributes to sustainable development, in general, and United Nations’ Sustainable Development Goals 3 and 4 more specifically through providing valuable insights into the prevalence and burden of ACD among pediatric individuals with T1D and propose means of combating these adverse effects. The generated insights will inform multiple health and care professions’ education (skilling, upskilling, and reskilling) and public health practices and lay the groundwork for future research and innovation aimed at improving adhesives’ design maximizing the overall value of the corresponding smart devices. This study calls for transdisciplinary collaborative endeavors between researchers, healthcare providers, biomedical and pharmaceutical companies, and regulatory bodies to develop hypoallergenic adhesives tailored for pediatric individuals with T1D. Furthermore, patch testing to identify specific allergens in adhesives would enable personalized interventions for affected individuals, thereby improving treatment adherence and optimizing glycemic control.

## Data Availability

The raw data supporting the conclusions of this article will be made available by the authors, without undue reservation.

## References

[1] DiMeglio LA Evans-Molina C Oram RA. Type 1 diabetes. Lancet. (2018) 391:2449–62. doi: 10.1016/S0140-6736(18)31320-5, PMID: 29916386 PMC6661119

[2] Castro MR. Mayo Clinic: The Essential Diabetes Book 3rd Edition: How to prevent, manage and live well with diabetes. Rochester, Minnesota, United States: Simon and Schuster (2022).

[3] Gregory GA Robinson TI Linklater SE Wang F Colagiuri S De Beaufort C. Global incidence, prevalence, and mortality of type 1 diabetes in 2021 with projection to 2040: a modelling study. Lancet Diabetes Endocrinol. (2022) 10:741–60. doi: 10.1016/S2213-8587(22)00218-2, PMID: 36113507

[4] Adhami S James S Paterson M Deeb A Craft J. Glycemic control of children and adolescents with type 1 diabetes in the United Arab Emirates: A narrative review. J Diabetes Endocrine Pract. (2025) 08(01):05–17. doi: 10.1055/s-0044-1801834

[5] Dabous RM Younes ZH Athamnah SB Hassoun AK. The effect of frequent diabetes self-management education on glucose control in patients with diabetes at the Dubai Diabetes Center in Dubai, United Arab Emirates. J Diabetes Endocrine Pract. (2019) 2:8–12.

[6] Navarro-Triviño F. Reacciones cutáneas a sensores de glucosa: presente y futuro. Actas Dermo-Sifiliográficas. (2021) 112:389–91. 10.1016/j.ad.2020.09.008 33220307

[7] Owen JL Vakharia PP Silverberg JI. The role and diagnosis of allergic contact dermatitis in patients with atopic dermatitis. Am J Clin Dermatol. (2018) 19:293–302. doi: 10.1007/s40257-017-0340-7, PMID: 29305764 PMC5948135

[8] Heinemann L Kamann S. Adhesives used for diabetes medical devices: a neglected risk with serious consequences? Los Angeles, CA: SAGE Publications Sage CA (2016) p. 1211–5. 10.1177/1932296816662949 PMC5094339 27566734

[9] Spiewak R. Patch testing for contact allergy and allergic contact dermatitis. Open Allergy J. (2008) 1:42–51. doi: 10.2174/1874838400801010042

[10] Li Y Li L. Contact dermatitis: classifications and management. Clin Rev Allergy Immunol. (2021) 61:245–81. doi: 10.1007/s12016-021-08875-0, PMID: 34264448

[11] Genève P Adam T Delawoevre A Jellimann S Legagneur C DiPatrizio M. High incidence of skin reactions secondary to the use of adhesives in glucose sensors or insulin pumps for the treatment of children with type 1 diabetes. Diabetes Res Clin Pract. (2023) 204:110922. doi: 10.1016/j.diabres.2023.110922, PMID: 37769906

[12] Lombardo F Salzano G Crisafulli G Panasiti I Alibrandi A Messina MF. Allergic contact dermatitis in pediatric patients with type 1 diabetes: an emerging issue. Diabetes Res Clin Pract. (2020) 162:108089. doi: 10.1016/j.diabres.2020.108089, PMID: 32087268

[13] Atlas D. International diabetes federation. IDF Diabetes Atlas. 7th Vol. 33. . Brussels, Belgium: International Diabetes Federation (2015).

[14] Nations U. Sustainable development 2015. Available online at: https://sdgs.un.org/goals (Accessed September 24, 2025).

[15] Al-Jayyousi R Mahfouz NA Otaki F Paulus A Czabanowska K Zaman Q. Investigating the learning value of early clinical exposure among undergraduate medical students in Dubai: a convergent mixed methods study. BMC Med Educ. (2025) 25:638. doi: 10.1186/s12909-025-07212-9, PMID: 40307797 PMC12044941

[16] Alameddine M AlGurg R Otaki F Alsheikh-Ali AA. Physicians’ perspective on shared decision-making in Dubai: a cross-sectional study. Hum Resour Health. (2020) 18:1–9. doi: 10.1186/s12960-020-00475-x, PMID: 32381007 PMC7206665

[17] Alameddine M Otaki F Bou-Karroum K Du Preez L Loubser P AlGurg R. Patients’ and physicians’ gender and perspective on shared decision-making: a cross-sectional study from Dubai. PloS One. (2022) 17:e0270700. doi: 10.1371/journal.pone.0270700, PMID: 36048748 PMC9436052

[18] Ibrahim NM Almarzouqi FI Al Melaih FA Farouk H Alsayed M AlJassim FM. Prevalence of asthma and allergies among children in the United Arab Emirates: A cross-sectional study. World Allergy Organ J. (2021) 14:100588. doi: 10.1016/j.waojou.2021.100588, PMID: 34703522 PMC8503660

[19] Abdalla N Al Meleh F Al Marzouqi F Al Jassim F Farouk H Alsayed M. Prevalence of asthma and allergies among children in the United Arab Emirates: A crosssectional study. South Yorkshire, United Kingdom: European Respiratory Society (2023).

[20] Mahmoud O Yosipovitch G Attia E. Burden of disease and unmet needs in the diagnosis and management of atopic dermatitis in the Arabic population of the Middle East. J Clin Med. (2023) 12:4675. doi: 10.3390/jcm12144675, PMID: 37510789 PMC10380694

[21] Ahrensbøll-Friis U Simonsen AB Zachariae C Thyssen JP Johansen JD. Contact dermatitis caused by glucose sensors, insulin pumps, and tapes: results from a 5-year period. Contact Dermatitis. (2021) 84:75–81., PMID: 32677709 10.1111/cod.13664

[22] Abdelmannan D AlBuflasa M Ajlouni H Zidan M Rahman F Farooqi MH. Institutional experience on the impact of glucagon-like peptide-1 agonists (GLP-1) on glycemic control and weight loss in patients with type 2 diabetes at the Dubai Diabetes Center, United Arab Emirates. Diabetes Res Clin Pract. (2024) 207:111045. doi: 10.1016/j.diabres.2023.111045, PMID: 38070546

[23] Organization WH. Core competencies in adolescent health and development for primary care providers: including a tool to assess the adolescent health and development component in pre-service education of health-care providers. Geneva, Switzerland: World Health Organization (2015).

[24] Organization WH. The adolescent health indicators recommended by the Global Action for Measurement of Adolescent Health: Guidance for monitoring adolescent health at country, regional and global levels. Geneva, Switzerland: World Health Organization (2024).

[25] Bawady N Aldafrawy O ElZobair EM Suliman W Alzaabi A Ahmed SH. Prevalence of overweight and obesity in type 2 diabetic patients visiting PHC in the Dubai health authority. Dubai Diabetes Endocrinol J. (2022) 28:20–4. doi: 10.1159/000519444

[26] Gazit V Tasher D Hanukoglu A Landau Z Ben-Yehuda Y Somekh E. Atopy in children and adolescents with insulin-dependent diabetes mellitus. Cytokines. (2008) 1:2., PMID: 19160942

[27] Popoviciu MS Kaka N Sethi Y Patel N Chopra H Cavalu S. Type 1 diabetes mellitus and autoimmune diseases: a critical review of the association and the application of personalized medicine. J Personalized Med. (2023) 13:422. doi: 10.3390/jpm13030422, PMID: 36983604 PMC10056161

[28] De Bock M Codner E Craig ME Huynh T Maahs DM Mahmud FH. ISPAD Clinical Practice Consensus Guidelines 2022: Glycemic targets and glucose monitoring for children, adolescents, and young people with diabetes. Pediatr Diabetes. (2022) 23:1270. doi: 10.1111/pedi.13455, PMID: 36537523 PMC10107615

[29] De Bock M Agwu JC Deabreu M Dovc K Maahs DM Marcovecchio ML. International society for pediatric and adolescent diabetes clinical practice consensus guidelines 2024: glycemic targets. Horm Res Paediatr. (2024) 97:546–54., PMID: 39701064 10.1159/000543266 PMC11854972

[30] Cameli N Silvestri M Mariano M Messina C Nisticò SP Cristaudo A. Allergic contact dermatitis, an important skin reaction in diabetes device users: a systematic review. Dermatitis. (2022) 33:110–5. doi: 10.1097/DER.0000000000000861, PMID: 35245221

[31] Pyl J Dendooven E Van Eekelen I Den Brinker M Dotremont H France A. Prevalence and prevention of contact dermatitis caused by FreeStyle Libre: a monocentric experience. Diabetes Care. (2020) 43:918–20. doi: 10.2337/dc19-1354, PMID: 32054722

[32] Passanisi S Galletta F Bombaci B Cherubini V Tiberi V Minuto N. Device-related skin reactions increase emotional burden in youths with type 1 diabetes and their parents. J Diabetes Sci Technol. (2024) 18:1293–9. doi: 10.1177/19322968241253285, PMID: 38804535 PMC11535255

[33] Von Kobyletzki LB Ulriksdotter J Sukakul T Aerts O Agner T Buhl T. Prevalence of dermatitis including allergic contact dermatitis from medical devices used by children and adults with Type 1 diabetes mellitus: A systematic review and questionnaire study. J Eur Acad Dermatol Venereol. (2024) 38:1329–46. doi: 10.1111/jdv.19908, PMID: 38400603

[34] Lombardo F Passanisi S Tinti D Messina MF Salzano G Rabbone I. High frequency of dermatological complications in children and adolescents with type 1 diabetes: a web-based survey. J Diabetes Sci Technol. (2021) 15:1377–81. doi: 10.1177/1932296820947072, PMID: 32757778 PMC8655296

[35] Podwojniak A Flemming J Tan IJ Ghani H Neubauer Z Jones A. Cutaneous adverse effects from diabetes devices in pediatric patients with type 1 diabetes mellitus: Systematic review. JMIR Dermatol. (2024) 7:e59824. doi: 10.2196/59824, PMID: 39622650 PMC11587996

[36] Kostner L Anzengruber F Guillod C Recher M Schmid-Grendelmeier P Navarini AA. Allergic contact dermatitis. Immunol Allergy Clinics North America. (2017) 37:141–52. doi: 10.1016/j.iac.2016.08.014, PMID: 27886903

[37] Al-Herz W. A systematic review of the prevalence of atopic diseases in children on the Arabian Peninsula. Med Principles Pract. (2018) 27:436–42. doi: 10.1159/000493267, PMID: 30149382 PMC6244036

[38] Al-Hammadi S Al-Maskari F Bernsen R. Prevalence of food allergy among children in Al-Ain city, United Arab Emirates. Int Arch Allergy Immunol. (2010) 151:336–42. doi: 10.1159/000250442, PMID: 19851075

[39] Redondo MJ Connor CG Ruedy KJ Beck RW Kollman C Wood JR. Pediatric diabetes consortium type 1 diabetes new onset (NeOn) study: factors associated with HbA1c levels one year after diagnosis. Pediatr Diabetes. (2014) 15:294–302. doi: 10.1111/pedi.12061, PMID: 23889707 PMC3858510

[40] Sayed A Alyafei F De Sanctis V Soliman A Elgamal M. Translating the HbA1c assay into estimated average glucose values in children and adolescents with type 1 diabetes mellitus. Acta Bio Med: Atenei Parmensis. (2018) 89:22., PMID: 30049928 10.23750/abm.v89i5.7357 PMC6179094

[41] Association AD. 13. Children and adolescents: standards of medical care in diabetes– 2020. Diabetes Care. (2020) 43:S163–S82. 10.2337/dc20-S013 31862756

[42] American Diabetes Association Professional Practice Committee. Children and adolescents: standards of care in diabetes—2025. Diabetes Care. (2025) 48:S283–305. doi: 10.2337/dc25-S014, PMID: 39651980 PMC11635046

[43] Passanisi S Salzano G Galletta F Aramnejad S Caminiti L Pajno GB. Technologies for type 1 diabetes and contact dermatitis: therapeutic tools and clinical outcomes in a cohort of pediatric patients. Front Endocrinol. (2022) 13:846137. doi: 10.3389/fendo.2022.846137, PMID: 35370980 PMC8965381

[44] Messer LH Berget C Beatson C Polsky S Forlenza GP. Preserving skin integrity with chronic device use in diabetes. Diabetes Technol Ther. (2018) 20:S254–S64. doi: 10.1089/dia.2018.0080, PMID: 29916740 PMC6011799

[45] Berg AK Nørgaard K Thyssen JP Zachariae C Hommel E Rytter K. Skin problems associated with insulin pumps and sensors in adults with type 1 diabetes: a cross-sectional study. Diabetes Technol Ther. (2018) 20:475–82. doi: 10.1089/dia.2018.0088, PMID: 29893593

[46] Moscrop R. Skin assessment and ongoing skincare with adhesive diabetes management devices. J Diabetes Nurs. (2023) 25.

[47] Cichoń M Trzeciak M Sokołowska-Wojdyło M Nowicki RJ. Contact dermatitis to diabetes medical devices. Int J Mol Sci. (2023) 24:10697. doi: 10.3390/ijms241310697, PMID: 37445875 PMC10341568

[48] Velasco-Amador J Prados-Carmona Á Navarro-Triviño F. Translated article] medical devices in patients with diabetes and contact dermatitis. Actas Dermo-Sifiliográficas. (2024) 115:T280–T7. doi: 10.1016/j.ad.2024.01.016, PMID: 38242434

[49] Cichoń M Myśliwiec M Trzeciak M. Role of acrylates in the development of contact dermatitis in diabetic patients—a Polish dermatology tertiary centre experience. Contact Dermatitis. (2024) 90:126–33., PMID: 37840370 10.1111/cod.14436

[50] Dahmani KMA Suliman M Hafidh K Beshyah SA. Epidemiology, technology, and professional perspectives on diabetes in the united arab emirate: A focused review. J Diabetes Endocrine Pract. (2024) 07(02):93–104. doi: 10.1055/s-0044-1786013

[51] VanArsdel PP Jr. Larson EB. Diagnostic tests for patients with suspected allergic disease: utility and limitations. Ann Internal Med. (1989) 110:304–12. doi: 10.7326/0003-4819-110-4-304, PMID: 2643916

[52] De Groot A Van Oers EM Ipenburg NA Rustemeyer T. Allergic contact dermatitis caused by glucose sensors and insulin pumps: A full review: Part 2. Case reports and case series, clinical features, patch test procedures, differentiation from irritant dermatitis, management of allergic patients and (proposed) legislation. Contact Dermatitis. (2025) 92:164–75. doi: 10.1111/cod.14697, PMID: 39600134 PMC11795346

